# A rare and an unusually delayed presentation of orbital actinomycosis following avulsion injury of the scalp

**DOI:** 10.4103/0301-4738.62653

**Published:** 2010

**Authors:** Vidya Hegde, Neelam Puthran, Mahesha S, Anupama B

**Affiliations:** Department of Ophthalmology, Yenepoya Medical College, Deralakatte, Mangalore, India

**Keywords:** Delayed presentation, head trauma, orbital actinomycosis, young adult

## Abstract

We report a rare case of orbital swelling presenting one year after head trauma. An initial fine needle aspiration cytology revealed it to be an infected organizing hematoma. However, broad-spectrum antibiotics did not resolve the infection and the orbital lesion continued to grow in size, as evaluated by magnetic resonance imaging. Incisional biopsies were done, which were reported as orbital actinomycosis. Patient has responded well to treatment with penicillin. This case is of interest due to the delayed presentation of an orbital complication of head trauma and the rare infection with actinomyces. It also highlights the importance of using appropriate antibiotics, as well as the need for long-term treatment.

Orbital actinomycosis has been reported uncommonly in literature.[1–4] The source of infection may be either from the oral cavity or paranasal sinuses or following trauma.[5,6] We report a case of this uncommon infection, which presented one year after avulsion injury of the scalp sustained in a road traffic accident.

## Case Report

A 20-year-old gentleman reported with inability to lift his right upper lid following the development of a painless swelling in the right brow region of two weeks' duration. There was no history of any diplopia, or visual disturbances and nor was there any concurrent dental, ear, nose, throat (ENT) or significant systemic illness. The patient had sustained deep lacerations of the scalp and forehead due to a road traffic accident a year earlier. There was no history of loss of consciousness, bleeding ENT or any visual disturbances at the time of injury. He had been treated elsewhere with wound suturing and antibiotics novoclox and metrogyll for five days, following which he recovered well and had remained symptom-free till the present illness.

**Figure 1:**
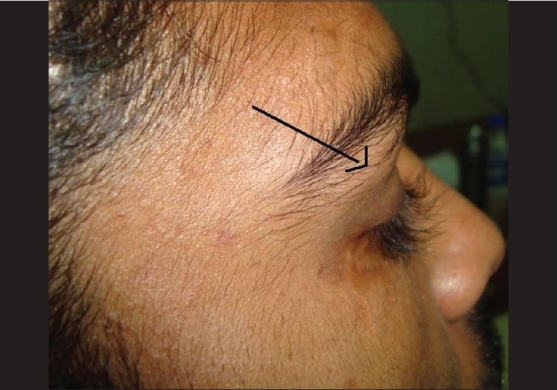
Arrow showing superotemporal orbital swelling

Clinical examination revealed a firm, non-tender, finely nodular swelling 2 cm × 4 cm in size, in the superotemporal region of the right upper lid, with an accompanying moderate grade mechanical ptosis. Skin over the swelling was normal and the mass seemed to be continuous with the supero-temporal orbital rim but not with the lacrimal gland [Fig. 1]. The frontalis muscle was acting normally. Both eyes were normal with a visual acuity of 20/20 in each eye. Exophthalmometric readings were normal. Dental, ENT and systemic examinations were also normal.

**Figure 2:**
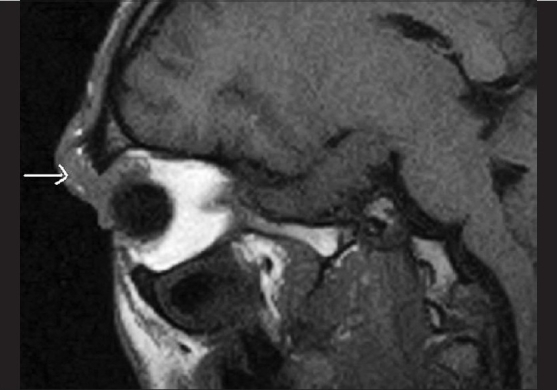
MRI sagital T1 showing soft tissue swelling in right periorbital region

**Figure 3:**
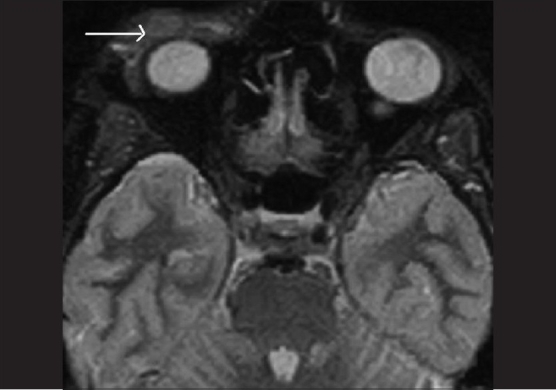
MRI fat suppressed STIR sequence showing the lesion

**Figure 4:**
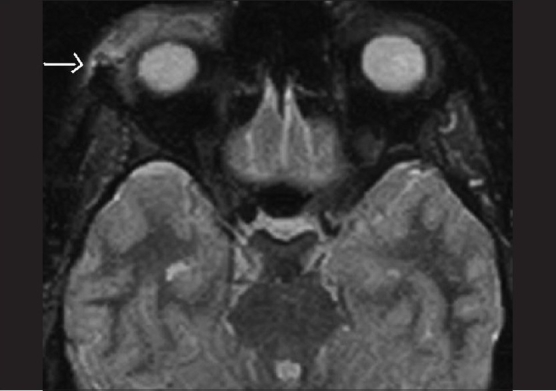
Repeat MRI fat suppressed STIR sequence showing increase in size of the lesion

Orbital X-rays revealed a soft tissue shadow anterior to the orbital margin. There were no bony abnormalities seen. Magnetic resonance imaging (MRI) revealed an ill-defined extraconal lesion at the superior and lateral aspect of the right orbit which seemed to involve the lacrimal gland. The radiologist's report suggested the presence of a pseudotumor orbit or lymphoma [Figs. 2 and 3]. Consequently, tissue samples from three different areas of the mass were obtained by fine needle aspiration cytology (FNAC), through both skin and conjunctival approaches. The FNAC was reported as organizing infected hematoma, although the infective organism was not identified. As treatment with intravenous cefotaxime 500 mg 12-hourly for five days produced only marginal improvement in the ptosis [Table 1] without any perceptible change in the lid mass, a repeat MRI was done a month later. This revealed an increase in the size of the lesion towards the orbit [Fig. 4].

**Table 1:** Ptosis evaluation

Right eye	Baseline measurements	After first course of antibiotics
Palpebral fissure height	5 mm	6 mm
MRD 1	2 mm	3 mm
LPS Function	5 mm	6 mm

**Figure 5:**
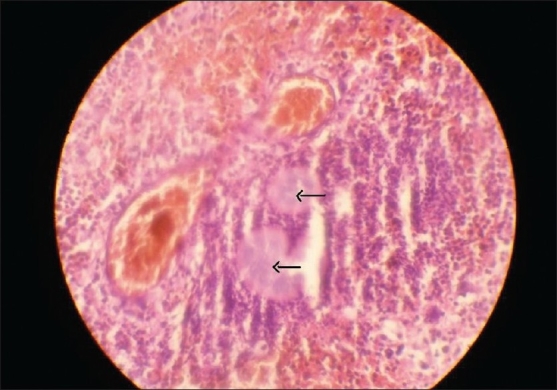
Photograph showing granulation tissue with arrows indicating Actinomycotic colonies (H & E × 40).

Patient was taken up for anterior orbitotomy through superior lid crease approach. A small old blood clot was removed and multiple incisional biopsies were obtained and sent for histopathological examination. Gram's staining showed gram-positive, non-acid-fast filaments with surrounding gram-negative reaction. The lesion was reported to be a case of actinomycosis infection [Fig. 5]. Patient has responded satisfactorily to intravenous penicillin G 30 lakh units 6-hourly for two weeks. For the prevention of recrudescence, patient is advised oral amoxicillin 500 mg 6-hourly for six months. He has been followed up for four months and has recovered fully.

## Discussion

Orbital lesions present a great challenge to ophthalmologists. In unilateral orbital swellings following trauma, the differential diagnosis includes subperiosteal hematomas and abscesses while pathologies like orbital pseudotumors and lymphangiomas unrelated to trauma are also to be kept in mind.

Subperiosteal hematomas are less common and generally occur in young adult males as a result of direct facial or orbital trauma,[7,8] or erosion of a vessel by orbital extension of an infectious process. They may also occur spontaneously at any age following sudden elevation of cranial venous pressure[9] thereby causing a tearing of subperiosteal vessels. The hematomas are frequently located in the orbital roof as the periosteum in this area is loosely attached.

In our case, although the initial suspicion was that of either an organized hematoma or post-traumatic hyperostosis of the orbital rim, the possibility of orbital lymphoma was also entertained due to the surface nodularity of the mass. While orbital X-rays ruled out hyperostosis, the MRI suggested a diagnosis of either pseudotumor orbit or lymphoma. However, FNAC reported the presence of an infected organized hematoma but the infecting organism could not be identified. Despite broad-spectrum antibiotics the swelling continued to grow. Incisional biopsies obtained through anterior orbitotomy revealed the presence of orbital actinomycosis.

Orbital actinomycosis is a very rare condition, with only a few cases reported in recent literature.[1–4] Actinomyces are filamentous, branching gram-positive bacilli normally inhabiting the oral cavity. They exhibit low pathogenicity and require mucosal barrier disruption to cause disease. Co-pathogens assist in the spread of infection, which occurs in a progressive manner, ignoring tissue planes. Infection is commonly caused by *Actinomyces israelli.*

While trauma is the commonest predisposing factor,[5] orbital involvement is often secondary to infections of the paranasal sinuses, or the ocular adnexal structures or even the infra-temporal fossa.[6] Although patients have presented varyingly with acute orbital abscess and painful ophthalmoplegia,[3,4] the usual presentation is as a painless proptosis with restricted extra-ocular movements, often mimicking a malignancy. In our patient, it is likely that the intra-orbital extent of the lesion was insufficient to produce globe displacement. The time of presentation may vary from within few weeks to a few months.[3,4] Our patient presented after an unusual delay of one year. In hindsight, it is felt that our patient probably had a primary inoculation at the time of scalp avulsion a year prior. The delayed presentation was due to the slow progression typical of actinomycotic infections.

Actinomycotic infections are best treated with intravenous penicillin G for two weeks, followed by oral penicillin or amoxicillin for six months. In case of penicillin allergy, tetracycline is used. Our patient showed a good response to intravenous crystalline penicillin and is presently on oral amoxicillin for six months to prevent recrudescence. This case highlights the confusing multitude of diagnoses, the rarity of orbital actinomycosis and the need for long-term antibiotic treatment.
